# Proteinuria Is Associated with the Development of Crohn’s Disease: A Nationwide Population-Based Study

**DOI:** 10.3390/jcm10040799

**Published:** 2021-02-16

**Authors:** Seona Park, Hyun Jung Lee, Kyung-Do Han, Hosim Soh, Jung Min Moon, Seung Wook Hong, Eun Ae Kang, Jong Pil Im, Joo Sung Kim

**Affiliations:** 1 Department of Internal Medicine and Liver Research Institute, Seoul National University College of Medicine, Seoul 03080, Korea; seven0526@naver.com (S.P.); hosimsoh@gmail.com (H.S.); moon_jm@naver.com (J.M.M.); jp-im@hanmail.net (J.P.I.); jooskim@snu.ac.kr (J.S.K.); 2 Department of Gastroenterology, Mediplex Sejong Hospital, Incheon 21080, Korea; 3 Department of Statistics and Actuarial Science, Soongsil University, Seoul 06978, Korea; hkd917@naver.com; 4 Department of Gastroenterology, Asan Medical Center, University of Ulsan College of Medicine, Seoul 05505, Korea; hswooki@gmail.com; 5 Department of Internal Medicine and Institute of Gastroenterology, Yonsei University College of Medicine, Seoul 03722, Korea; cheerea@gmail.com

**Keywords:** claims data, incidence, proteinuria, Crohn’s disease

## Abstract

Background and Aims: The impact of proteinuria and its severity on the incidence of inflammatory bowel disease (IBD) has not yet been studied. We aimed to determine the association between proteinuria measured by urine dipstick tests and the development of IBD. Methods: This nationwide population-based study was conducted using the Korean National Health Insurance Service (NHIS) database. A total of 9,917,400 people aged 20 years or older who had undergone a national health examination conducted by the NHIS in 2009 were followed up until 2017. The study population was classified into four groups—negative, trace, 1+, and ≥ 2+—according to the degree of proteinuria measured by the urine dipstick test. The primary endpoint was newly diagnosed IBD, Crohn’s disease (CD), or ulcerative colitis (UC) during the follow-up period. Results: Compared with the dipstick-negative group, the incidence of CD significantly increased according to the degree of proteinuria (adjusted hazard ratio [aHR] with 95% confidence interval [CI], 1.01 [0.703–1.451], 1.515 [1.058–2.162], and 2.053 [1.301–3.24] in the trace, 1+, and ≥ 2+ dipstick groups, respectively; *p* for trend 0.007). However, there was no significant difference in the incidence of UC according to the degree of proteinuria (aHR with 95% CI, 1.12 [0.949–1.323], 0.947 [0.764–1.174], and 1.009 [0.741–1.373] in the trace, 1+, and ≥ 2+ dipstick groups, respectively; *p* for trend 0.722). In the subgroup analysis, dipstick-positive proteinuria independently increased the incidence of CD regardless of the subgroup. However, dipstick-positive proteinuria was associated with the risk of UC in those with diabetes mellitus and not in those without diabetes mellitus (aHR, 1.527 vs. 0.846; interaction *p*-value 0.004). The risk of CD was increased or decreased according to proteinuria changes but not associated with the risk of UC. Conclusion: Proteinuria, measured by the dipstick test, is strongly associated with the development of CD.

## 1. Introduction

Inflammatory bowel disease (IBD), including Crohn’s disease (CD) and ulcerative colitis (UC), is a chronic, immune-related, progressive disease characterized by repeated improvements and deteriorations. Complex interactions between genetic elements, environmental factors, and the gut microbiome lead to the pathogenesis of IBD [1]. The prevalence and incidence of IBD are high in North America and Europe and relatively low in Asia [2] but, due to rapid socioeconomic changes, its incidence has more than doubled in recent decades in East Asia, including Japan, Korea, and Hong Kong [3]. Accordingly, IBD is becoming a global disease and its direct and indirect costs are also increasing enormously, which will become a major health problem in future societies [4]. Therefore, it is necessary to identify and screen for risk factors that can be modified before the onset of IBD.

Proteinuria is a marker of kidney damage that is associated with a rapid deterioration in renal function as the proteinuria severity increases [5]. Proteinuria also predicts patients at increased risk of adverse clinical outcomes regardless of the estimated glomerular filtration rate (eGFR) [6]. In addition, proteinuria has been regarded as a manifestation of endothelial dysfunction and systemic inflammation [7] and has recently been linked to the risk of chronic systemic inflammatory diseases [8,9,10,11,12,13]. However, there is no evidence on the impact of proteinuria on the incidence of IBD. Therefore, using a nationwide database, we examined the association of proteinuria with the risk of IBD.

## 2. Material and Methods

### 2.1. Data Source and Study Population

This nationwide cohort study was performed using data from the National Health Insurance Service (NHIS), which is mandatory for all healthcare service providers and citizens in South Korea. The NHIS is a single payment program supported by the Korean government and, because the NHIS database covers the entire Korean population, it is suitable for population-based nationwide studies [14]. The NHIS database includes demographic information, disease codes, procedural codes, inpatient and outpatient care details, and information on prescribed drugs. These data are encrypted to prevent the recognition of personal information. The NHIS provides standardized health checkups to detect diseases early and provide subsequent medical benefits to the insured and recommends that patients undergo a health checkup at least once every 2 years. The health checkup includes height, weight, body mass index (BMI), and blood pressure measurements and basic laboratory tests, including urinalysis, fasting blood glucose, total cholesterol, creatinine, and eGFR. Past medical history, social history including alcohol consumption and smoking status, and physical activity are collected from self-reported questionnaires. In the above database, we evaluated data from Koreans older than 19 years old (range 20–110 years) who received at least one health checkup provided by the NHIS from January 2009 to December 2009. Those diagnosed with IBD from January 2004 to the time of the health checkup (index date) and those diagnosed with IBD within 1 year after the index date (lag period) were excluded. Individuals with missing data for any variable were also excluded. The remaining individuals were then tracked from the index date until December 2017. This study was approved by Seoul National University College of Medicine-Seoul National University Hospital Institutional Review Board (SNUCM-SNUH IRB) on 24 March 2017 (H-1703-107-840).

### 2.2. Dipstick Urinalysis and Study Endpoints

Proteinuria was measured by the urine dipstick test. Although the urine protein-to-creatinine ratio (PCR) and urine albumin-to-creatinine ratio (ACR) are used as quantitative tests for proteinuria, they are expensive to use for public health screening and require a long time for result confirmation. The urine dipstick test is a relatively simple, fast, and inexpensive method for screening proteinuria in public healthcare systems. In the urine dipstick test, the degree of proteinuria is measured as negative, trace, 1+, 2+, 3+, or 4+ according to the proteinuria concentration-dependent color difference. The study population was classified into four groups according to the degree of proteinuria: negative, trace, 1+, and ≥ 2+.

The primary outcome of this study was newly developed IBD. In 2006, the Korean government established the Rare and Intractable Disease (RID) registration system to improve medical security by exempting patients from the burden of medical treatment for severe RIDs that require expensive and high-level procedures. Patients enrolled in the RID registration system are only charged 10% of the medical fees associated with the disease. CD and UC are classified as RIDs in Korea. Patients enrolled in the RID registration system are assigned a special code, V. The RID code is highly reliable because the strict diagnostic standards based on the various examination items and criteria provided by the NHIS must be verified by a qualified Korean physician [15]. In this study, we identified incident IBD using both International Classification of Disease, Tenth Revision (ICD-10) codes (CD, K50; UC, K51) and V codes (CD, V130; UC, V131), as in previous studies [16,17,18,19]. To confirm the accuracy of the incident IBD diagnosed by both V and ICD-10 codes, the medical records of IBD patients identified with both V and ICD-10 codes from January 2010 to March 2013 were retrospectively reviewed at Seoul National University Hospital, a tertiary referral hospital in Korea, and sensitivity analysis was performed. The diagnostic sensitivity of CD and UC was 94.5% and 96.4%, respectively.

### 2.3. Covariates

Information on demographics (age, sex, and residential area) and health checkup items (height, weight, BMI, blood pressure measurements, drinking behavior, smoking history, and exercise and baseline laboratory findings, including fasting blood glucose and total cholesterol) in the study population was evaluated as baseline characteristics. Individuals were classified as nonsmokers, former smokers, and current smokers according to smoking status. Subjects who consumed at least 30 g of alcohol per day were defined as heavy drinkers. The regular exercise group performed high-intensity exercise for at least 20 min or moderate-intensity exercise for at least 30 min at a time at least once a week. Baseline comorbidities, including diabetes mellitus (DM), hypertension, and hyperlipidemia, were also defined as in previous studies [16,17,18,19,20]. DM was defined as fasting blood glucose ≥ 126 mg/dL or as ICD-10 code E11-14 with a prescription for anti-diabetic medications. Hypertension was defined as a systolic/diastolic blood pressure ≥ 140/90 mmHg or as ICD-10 code I10-13 or I15 with a prescription of anti-hypertensive agents. Dyslipidemia was defined as fasting serum total cholesterol ≥ 240 mg/dL or as ICD-10 code E78 and a prescription for lipid-lowering drugs. eGFR was estimated using the Modification of Diet in Renal Disease study equation [21]. Dipstick-positive proteinuria was defined as a score of 1+ or more in the urine dipstick test.

### 2.4. Statistical Analysis

Data are presented as mean with standard deviation for continuous variables and as number with percentage for categorical variables. The incidence rate of IBD according to the degree of proteinuria was calculated as the total number of incident IBD cases divided by the total person-years and expressed as number per 100,000 person-years. The cumulative incidences of IBD according to the degree of proteinuria were compared using the Kaplan–Meier method and the log-rank test. To examine the association between proteinuria and the risk of IBD, we used the Cox proportional hazards model and presented the results as hazard ratios (HRs) with 95% confidence intervals (CIs). *p* < 0.05 was considered statistically significant. SAS version 9.4 (SAS Institute, Cary, NC, USA) and R version 3.2.3 (The R Foundation for Statistical Computing, Vienna, Austria) were used for statistical analysis.

## 3. Results

### 3.1. Baseline Characteristics of the Study Population

A total of 9,917,400 individuals were enrolled in this study. They were divided into four groups according to the degree of proteinuria: negative (*n* = 9,444,344, 95.2%), trace (*n* = 224,793, 2.3%), 1+ (167,324, 1.7%), and ≥ 2+ (80,939, 0.8%). The baseline characteristics of the study population by proteinuria are shown in Table 1. Severity of proteinuria was positively correlated with age, smoking history, and heavy drinking, as well as with BMI, weight, and creatinine level (all *p* < 0.001). In addition, this group had higher blood pressure, higher fasting blood glucose and total cholesterol levels, and more co-morbidities including hypertension, DM, and dyslipidemia (all *p* < 0.001). However, the rates of current smoking and regular exercise were significantly lower, in addition to height and eGFR levels, in people with higher proteinuria levels (all *p* < 0.001).

### 3.2. Incidence and Risk of IBD According to Proteinuria Degree on Dipstick

**Figure 1:**
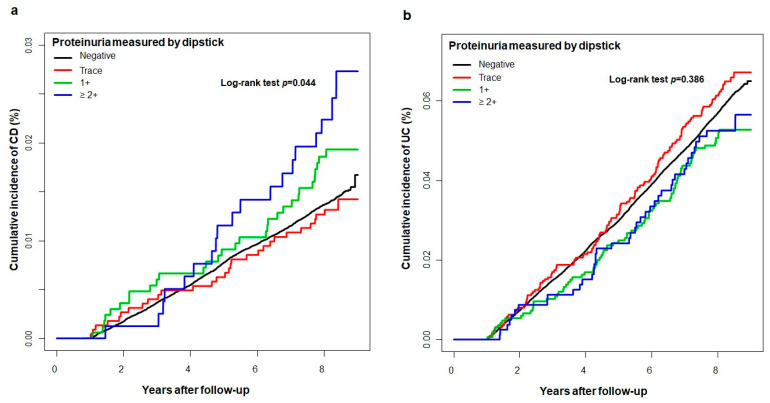
Kaplan-Meier curve for the cumulative incidence of Crohn’s disease (**a**) and (**b**) ulcerative colitis according to the degree of proteinuria measured by dipstick. CD, Crohn’s disease; UC, ulcerative colitis.

During a median follow-up of 9.3 years (interquartile range 9.1–9.6), the incidence rates of CD were 1.7, 1.6, 2.3, and 2.9 per 100,000 person-years in the negative, trace, 1+, and ≥ 2+ proteinuria groups, respectively. A higher degree of proteinuria on the dipstick test was associated with an increased risk of CD development (crude HR, 1.72; 95% CI, 1.094–2.706 in the ≥ 2+ proteinuria group, *p* = 0.034; Table 2 and Figure 1a). After adjustment for age, sex, income level, smoking status, alcohol consumption status, exercise level, body mass index, eGFR, and comorbidities (DM, hypertension, and dyslipidemia), the risk of CD development was also significantly higher as the degree of proteinuria increased compared with the negative proteinuria group (adjusted HRs [aHRs], 1.01, 1.515, and 2.053 in the trace, 1+, and ≥ 2+ proteinuria groups, respectively, *p* for trend 0.007; Model 3 in Table 2). The incidence rates of UC were 7.2, 7.7, 6.2, and 6.3 per 100,000 person-years in the negative, trace, 1+, and ≥ 2+ proteinuria groups, respectively. However, the incidence and risk of UC development were not significantly different according to the degree of proteinuria (Table 2 and Figure 1b).

### 3.3. Subgroup Analysis of the IBD Risk in Dipstick-Positive Proteinuria

In the subgroup analysis, we identified the effects of dipstick-positive proteinuria (≥ 1+ proteinuria) on the development of CD and UC according to subgroups classified by age, sex, eGFR, regular exercise, and presence of obesity, DM, and hypertension (Table 3).

The effect of dipstick-positive proteinuria on the development of CD was not significantly different among all subgroups. Dipstick-positive proteinuria increased the risk of CD regardless of age, sex, eGFR, regular exercise, obesity, DM, or hypertension. However, dipstick-positive proteinuria was associated with the increased risk of UC in individuals with DM, but not in those without DM (aHR, 1.527 vs. 0.846; interaction *p*-value 0.004). The effect of dipstick-positive proteinuria on the risk of UC was not significantly different in the other subgroups except the DM subgroup.

### 3.4. Change in Proteinuria on the Dipstick Test and Risk of IBD

**Figure 2:**
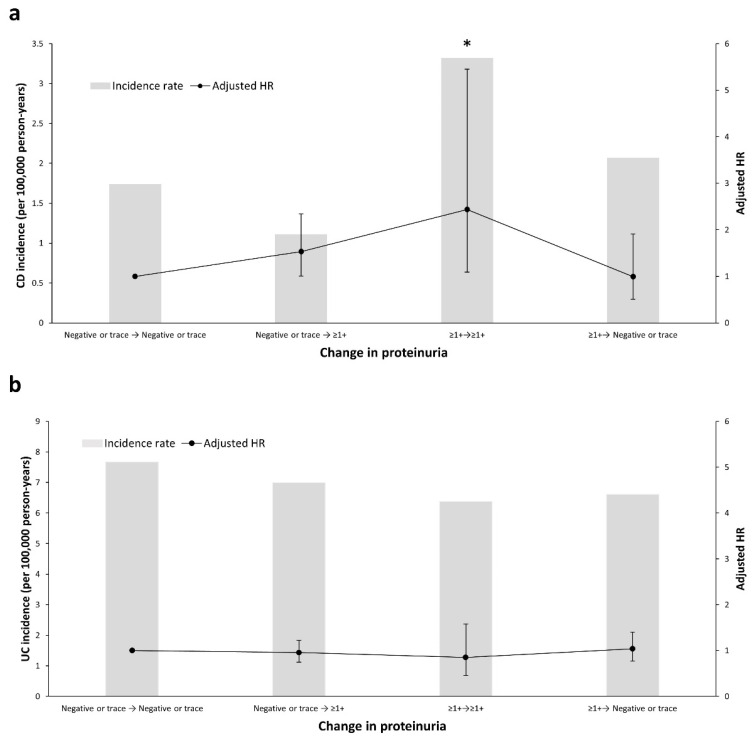
Incidence and risk of (**a**) Crohn’s disease and (**b**) ulcerative colitis according to changes in proteinuria measured by dipstick. Hazard ratios were adjusted for age, sex, income level, smoking status, alcohol consumption status, exercise level, body mass index, estimated glomerular filtration rate, diabetes mellitus, hypertension, and dyslipidemia. The left Y axis and columns indicate the incidence rate, the right Y axis and line indicate the adjusted HR, and the error bars indicate the 95% confidence interval. * *p* < 0.05. CD, Crohn’s disease; HR, hazard ratio; UC, ulcerative colitis.

Of a total of 9,917,400 participants, 6,078,732 (61.3%) underwent another health checkup within 2 years after the baseline checkup. Most of the people (*n* = 5,809,318, 95.6%) who initially had dipstick-negative or trace results showed the same results at follow-up. Compared with these, there was an increased tendency of CD development among individuals who had dipstick-positive results during follow-up, although not statistically significant (aHR, 1.513; 95% CI, 0.989–2.316, negative or trace → ≥ 1+ proteinuria) (Figure 2a). In addition, the risk of developing CD was highest in people with persistent dipstick-positive proteinuria during follow-up (aHR, 2.364; 95% CI, 1.053–5.306, ≥ 1+ → ≥ 1+ proteinuria). However, the reduced dipstick-positive proteinuria at follow-up was not accompanied by a reduced risk of developing CD during follow-up (aHR, 0.977; 95% CI, 0.506–1.886, ≥ 1+ → negative or trace). In contrast to CD, the association between the change in proteinuria and UC risk was not significant (Figure 2b).

## 4. Discussion

This is the first study to demonstrate the effect of proteinuria measured by dipstick tests on the development of IBD. In this nationwide population-based cohort study using health screening data on approximately 10,000,000 members of the general population, we found that dipstick-positive proteinuria was significantly associated with increased risk of CD development, but not UC development, after adjusting for potential confounding factors. As the degree of proteinuria increased, the HR of CD development also increased, and individuals with persistent proteinuria during follow-up had the highest risk of CD development. Therefore, dipstick-positive proteinuria may be a predictive risk factor for the development of CD.

ACR or PCR measurement in a spot urine sample is recommended as an appropriate method for the detection of proteinuria. However, these tests are time-consuming and costly. Accordingly, the urine dipstick test is widely accepted as an initial screening tool in the general population [22,23]. Compared with ACR or PCR, the urine dipstick test shows high sensitivity and specificity in detecting proteinuria. In particular, 1+ or greater dipstick results identify ACR ≥ 300 mg/g with a sensitivity of 95% to 98% and specificity of 92% [22,24]. Therefore, dipstick-positive proteinuria, defined as 1+ or greater in this study, can reliably identify significant proteinuria.

In previous studies, proteinuria was identified as a sensitive marker of progressive renal dysfunction [25]. However, it is not only a reflection of renal pathology, but it is also associated with increased vascular permeability [7,26]. When endothelial dysfunction is caused by generalized damage to the endothelial glycocalyx covering the luminal surface of the vascular endothelium, it appears as an increase in systemic microvascular permeability with proteinuria at the glomerular level [27]. Isolated proteinuria might be an early reflection of increased systemic microvascular permeability and a marker of endothelial dysfunction, which may be associated with chronic low-grade systemic inflammation [28,29]. Prior studies have demonstrated that low-grade systemic inflammation, as measured by circulating C-reactive protein in patients with asymptomatic proteinuria, is increased compared to controls and negatively correlated with microvascular endothelial function determined by acetylcholine iontophoresis [7]. In addition, proteinuria has been identified to be associated with the development of many inflammatory conditions, including DM, infectious diseases [13,27], Parkinson’s disease [8], metabolic syndrome [12], and nonalcoholic fatty liver disease [9].

Endothelial dysfunction and increased vascular permeability play crucial roles in the pathogenesis of IBD [30,31,32]. This may result in the upregulation of cellular adhesion molecules, impaired barrier integrity, increased leukocyte diapedesis, vascular smooth muscle tone, and pro-coagulant status, leading to an inflammatory process in the gastrointestinal tract [33,34]. In IBD, the expressions of intercellular adhesion molecule-1, vascular cell adhesion molecule-1, and mucosal addressin cell adhesion molecule 1 (MAdCAM-1) are upregulated, increasing leukocyte recruitment. MAdCAM-1 interacts with the α4β7 integrins of naive CD4+ T cells and recruits these cells [33,35]. In addition, pro-inflammatory cytokines such as interleukin (IL)-1, tumor necrosis factor (TNF)-α, nitric oxide, vascular endothelial growth factor (VEGF), and IL-6 are increased in IBD, altering vascular permeability [33,36,37]. These findings suggest that chronic inflammation and endothelial dysfunction might contribute to potential mechanisms underlying the relationship between proteinuria and IBD. Dietary factors have also been linked to the pathogenesis of IBD via alterations in the microbiome, gut barrier function, and immunity [38]. Dietary patterns that can affect proteinuria, such as the Western diet and high animal protein, have also been related to increased risk of IBD [39]. Further study to evaluate the role of diet in the association between proteinuria and IBD is required.

Meanwhile, we found that a higher degree of proteinuria increased the risk of CD development but did not increase that of UC. Although the mechanism by which proteinuria affects the development of CD and UC in different ways is unknown, the inflammation in UC is typically restricted to the mucosa, whereas CD shows extensive transmural inflammation. Therefore, the systemic inflammatory response reflected by proteinuria appears to be more related to the development of CD than UC. In previous studies comparing inflammatory markers between patients with CD and UC, the higher production of C-reactive protein was observed in patients with CD than in patients with UC [40,41,42]. Serum IL-6 [43] and TNF-α [44] concentrations and TNF-α protein [45] and mRNA [46] expression in mucosal tissues were also significantly higher in patients with CD than in patients with UC. In addition, vascular etiology seems to be more involved in the development of CD. VEGF, which plays a role in increasing vascular permeability, is distinctly increased in patients with CD compared with UC patients and controls [47]. Therefore, dipstick-positive proteinuria, which is thought to reflect vascular permeability and systemic inflammation, may be more related to the development of CD than UC. In this study, dipstick-positive proteinuria increased the risk of CD as the severity of proteinuria increased. The effect did not differ according to age, sex, and co-morbidities, and recent studies have also reported a higher risk of cardiovascular diseases and DM even in young patients with CD [17,48]. Our results suggest that proteinuria can independently predict the risk of CD, apart from previously known risk factors. Further studies are needed to clarify the link between proteinuria and IBD subtypes.

Although we could not accurately account for the cause of the changes in proteinuria in this study, we found that the risk of CD decreased with a reduction in dipstick-positive proteinuria. In addition, we also found that the risk of CD development increased 1.5-fold when dipstick-positive proteinuria occurred, even with negative or trace results at baseline. Furthermore, the risk of CD development increased 2.3-fold when dipstick-positive proteinuria was repeatedly detected. Our results indicate that measurement of not only baseline proteinuria but also its serial change is important in clinical practice.

This study has several limitations. A major limitation is that because of its retrospective study design, it was not possible to determine the causal relationship accurately. Second, because information on the severity of incident IBD was unavailable in the NHIS database, we could not assess how the degree of proteinuria affects the severity of intestinal inflammation. In addition, we could not acquire data regarding dietary patterns and family history of individuals in the IBD database, as the NHI provides only non-identifiable information. Third, data on prescription drug use (angiotensin-converting enzyme inhibitors, angiotensin receptor blockers, and statins) that could affect proteinuria could not be evaluated in this study. Therefore, further study is mandatory to determine the risk of IBD development according to changes in medications known to cause proteinuria. Despite these limitations, considering the increased prevalence and incidence of IBD in Korea [49], and anticipating a future burden of disease, our study has implications toward examining modifiable risk factors of IBD.

## 5. Conclusions

In conclusion, dipstick-positive proteinuria predicted an increased risk of CD development, but not that of UC. In addition, the risk of CD development was modified by changes in proteinuria. Therefore, our data suggest that dipstick-positive proteinuria has the potential to be used as a surrogate marker of CD development.

## Figures and Tables

**Table 1:** Baseline characteristics of the study population.

Characteristics	Proteinuria Measured by Dipstick				*p*-Value
	Negative	Trace (±)	1+	≥ 2+	
No. (%)	9,444,344 (95.2)	224,793 (2.3)	167,324 (1.7)	80,939 (0.8)	
Age, years ^†^	46.9 ± 14.0	48.9 ± 14.2	51.1 ± 14.5	53.2 ± 14.3	<0.001
20–64	8,243,115 (87.3)	189,309 (84.2)	133,790 (80.0)	61,528 (76.0)	<0.001
65–110	1,201,229 (12.7)	35,484 (15.8)	33,534 (20.0)	19,411 (24.0)	
Male	5,177,903 (54.8)	121,349 (54.0)	90,214 (53.9)	45,386 (56.1)	<0.001
Urban residence	4,340,165 (46.0)	109,178 (48.6)	80,618 (48.2)	37,299 (46.1)	<0.001
Smoking status					<0.001
Nonsmokers	5,614,771 (59.5)	134,436 (59.8)	100,081 (59.8)	47,878 (59.2)	
Former smokers	1,351,140 (14.3)	33,116 (14.7)	25,809 (15.4)	13,498 (16.7)	
Current smokers	2,478,433 (26.2)	57,241 (25.5)	41,434 (24.8)	19,563 (24.2)	
Heavy drinker ^‡^	643,540 (6.8)	16,784 (7.5)	13,153 (7.9)	6518 (8.1)	<0.001
Regular exercise ^§^	4,870,116 (51.6)	114,758 (51.1)	81,648 (48.8)	37,829 (46.7)	<0.001
BMI, kg/m^2 †^	23.7 ± 3.2	24.0 ± 3.4	24.3 ± 3.5	24.6 ± 3.7	<0.001
Height, cm ^†^	163.9 ± 9.2	163.6 ± 9.1	163.0 ± 9.1	162.6 ± 9.1	<0.001
Weight, kg ^†^	63.9 ± 11.6	64.4 ± 11.9	64.7 ± 12.1	65.3 ± 12.5	<0.001
Systolic blood pressure, mmHg ^†^	122.2 ± 14.8	124.1 ± 16.1	126.5 ± 17.0	129.8 ± 18.1	<0.001
Diastolic blood pressure, mmHg^†^	76.2 ± 9.9	77.2 ± 10.6	78.43 ± 11.0	79.9 ± 11.4	<0.001
Fasting glucose, mg/dL ^†^	96.70 ± 21.9	101.7 ± 29.5	107.7 ± 37.0	115.2 ± 44.8	<0.001
Total cholesterol, mg/dL ^†^	194.8 ± 36.4	197.6 ± 38.3	199.6 ± 40.2	202.4 ± 43.4	<0.001
Creatinine, mg/dL ^†^	1.0 ± 0.9	1.1 ± 1.1	1.1 ± 1.0	1.2 ± 1.0	<0.001
eGFR, mL/min/1.73 m^2 †^	88.6 ± 44.5	85.0 ± 31.5	82.8 ± 33.9	77.4 ± 35.4	<0.001
Comorbidity					
Diabetes mellitus	2,353,771 (24.9)	75,690 (33.7)	72,603 (43.4)	45,808 (56.6)	<0.001
Hypertension	767,049 (8.1)	32,227 (14.3)	36,117 (21.6)	25,807 (31.9)	<0.001
Dyslipidemia	1,683,466 (17.8)	51,939 (23.1)	47,233 (28.2)	29,879 (36.9)	<0.001

BMI, body mass index; eGFR, estimated glomerular filtration rate; No., number. ^†^ Mean ± standard deviation. ^‡^ Defined as a person who consumed at least 30 g of alcohol per day. ^§^ Defined as high-intensity exercise for at least 20 min or moderate-intensity exercise for at least 30 min at a time at least once a week.

**Table 2:** Incidence and risk of Crohn’s disease and ulcerative colitis according to the degree of proteinuria measured by dipstick.

IBD	Proteinuria Measured by Dipstick				*p* for Trend
	Negative	Trace(±)	1+	≥ 2+	
Crohn’s disease					
CD cases (*n*)	1343	30	31	19	
Person-years	78,055,660	1,846,270	1,360,989	645,798	
CD incidence (per 100,000 person-years)	1.7	1.6	2.3	2.9	
Crude HR (95% CI)	1 (reference)	0.946 (0.659–1.358)	1.328 (0.93–1.895)	1.72 (1.094–2.706)	0.095
*p*-value		0.593	0.288	0.034	
Model 1 ^†^ HR (95% CI)	1 (reference)	0.99 (0.689–1.421)	1.454 (1.018–2.077)	1.949 (1.238–3.066)	0.016
*p*-value		0.778	0.049	0.021	
Model 2 ^‡^ HR (95% CI)	1 (reference)	1.01 (0.704–1.451)	1.515 (1.061–2.164)	2.056 (1.306–3.235)	0.006
*p*-value		0.879	0.039	0.011	
Model 3 ^§^ HR (95% CI)	1 (reference)	1.01 (0.703–1.451)	1.515 (1.058–2.162)	2.053 (1.301–3.24)	0.007
*p*-value		0.871	0.035	0.013	
Ulcerative colitis					
UC cases (*n*)	5634	143	85	41	
Person-years	78,040,796	1,845,890	1,360,805	645,715	
UC incidence (per 100,000 person-years)	7.2	7.7	6.2	6.3	
Crude HR (95% CI)	1 (reference)	1.074 (0.91–1.268)	0.868 (0.701–1.075)	0.886 (0.652–1.204)	0.16
*p*-value		0.367	0.115	0.277	
Model 1 ^†^ HR (95% CI)	1 (reference)	1.085 (0.919–1.281)	0.882 (0.712–1.093)	0.897 (0.66–1.22)	0.172
*p*-value		0.339	0.128	0.268	
Model 2 ^‡^ HR (95% CI)	1 (reference)	1.1 (0.932–1.299)	0.911 (0.735–1.128)	0.938 (0.689–1.275)	0.363
*p*-value		0.256	0.223	0.412	
Model 3 ^§^ HR (95% CI)	1 (reference)	1.12 (0.949–1.323)	0.947 (0.764–1.174)	1.009 (0.741–1.373)	0.722
*p*-value		0.185	0.370	0.682	

CD, Crohn’s disease; CI, confidence interval; HR, hazard ratio; UC, ulcerative colitis. ^†^ Model 1: age and sex adjusted. ^‡^ Model 2: model 1 + income level, smoking status, alcohol consumption status, exercise level, body mass index, and estimated glomerular filtration rate adjusted. ^§^ Model 3: model 2 + diabetes mellitus, hypertension, and dyslipidemia adjusted.

**Table 3:** Subgroup analysis of the risk of Crohn’s disease and ulcerative colitis among people with dipstick-positive proteinuria.

Subgroup	Crohn’s Disease		Ulcerative Colitis	
	Adjusted HR ^†^ (95% CI)	*p* for Interaction	Adjusted HR ^†^ (95% CI)	*p* for Interaction
Age, years				
20–64	1.732 (1.27–2.361)	0.828	0.903 (0.741–1.101)	0.068
65–110	1.459 (0.708–3.003)		1.33 (0.886–1.996)	
Sex				
Male	1.515 (1.032–2.224)	0.099	0.984 (0.794–1.221)	0.489
Female	2.099 (1.38–3.194)		0.899 (0.657–1.231)	
eGFR, mL/min/1.73 m^2^				
≥ 90	1.703 (1.063–2.728)	0.340	0.945 (0.693–1.287)	0.552
60–90	1.46 (0.952–2.238)		0.923 (0.721–1.181)	
< 60	2.217 (1.109–4.431)		1.303 (0.818–2.078)	
Regular exercise ^‡^				
No	1.726 (1.162–2.564)	0.938	1.102 (0.869–1.397)	0.143
Yes	1.633 (1.083–2.462)		0.831 (0.635–1.086)	
Obese ^§^				
No	1.765 (1.255–2.452)	0.711	0.987 (0.794–1.228)	0.912
Yes	1.482 (0.863–2.546)		0.922 (0.68–1.251)	
Diabetes mellitus				
No	1.532 (1.099–2.135)	0.171	0.846 (0.687–1.041)	0.004
Yes	2.28 (1.281–4.057)		1.527(1.073–2.174)	
Hypertension				
No	1.617 (1.111–2.355)	0.572	0.936(0.738–1.188)	0.720
Yes	1.751 (1.127–2.719)		0.987(0.755–1.288)	

CI, confidence interval; HR, hazard ratio; eGFR, estimated glomerular filtration rate. ^†^ Age, sex, income level, smoking status, alcohol consumption status, exercise level, body mass index, estimated glomerular filtration rate, diabetes mellitus, hypertension, and dyslipidemia adjusted. ^‡^ Defined as high-intensity exercise for at least 20 min or moderate-intensity exercise for at least 30 min at a time at least once a week. ^§^ Defined as a body mass index ≥ 25 kg/m^2^.

## Abbreviations

aHR, adjusted hazard ratio; ACR, albumin-to-creatinine ratio; BMI, body mass index; CI, confidence interval; CD, Crohn’s disease; DM, diabetes mellitus; eGFR, estimated glomerular filtration rate; HR, hazard ratio; IBD, inflammatory bowel disease; IL, interleukin; ICD-10, International Classification of Disease, Tenth Revision; MAdCAM, mucosal addressin cell adhesion molecule; NHIS, National Health Insurance Service; PCR, protein-to-creatinine ratio; RID, rare and intractable disease; TNF, tumor necrosis factor; UC, ulcerative colitis; VEGF, vascular endothelial growth factor.
